# Increasing incidence of anaphylaxis in Hong Kong from 2009 to 2019—discrepancies of anaphylaxis care between adult and paediatric patients

**DOI:** 10.1186/s13601-020-00355-6

**Published:** 2020-11-19

**Authors:** Philip Hei Li, Agnes S. Y. Leung, Rebecca M. Y. Li, Ting-fan Leung, Chak-sing Lau, Gary W. K. Wong

**Affiliations:** 1 Division of Rheumatology and Clinical Immunology, Department of Medicine, Queen Mary Hospital, The University of Hong Kong, Hong Kong, China; 2 Department of Paediatrics, Prince of Wales Hospital, The Chinese University of Hong Kong, Hong Kong, China

**Keywords:** Allergy, Anaphylaxis, Adrenaline autoinjector, Epidemiology, Incidence

## Abstract

**Background:**

Anaphylaxis has been increasing in developed countries but there is very little published data on the burden of anaphylaxis and the pattern of adrenaline autoinjector (AAI) prescription from Asia. We aim to determine the incidence rates of anaphylaxis and prescription rates of AAI over the past decade in Hong Kong.

**Methods:**

Using a centralized electronic database of Hong Kong’s sole public-funded healthcare provider, we obtained and analysed all patients between 2009 and 2019 with physician-reported diagnosis of anaphylaxis. Incidence rates were calculated using population statistics as the denominator. Patients’ prescriptions on discharge were collected to determine the AAI prescription rates.

**Results:**

The overall 10-year estimated incidence rate of anaphylaxis was 3.57 per 100,000 person-years. An increasing trend over time across both paediatric and adult populations from 2009 to 2014 was found, which remained stable until 2019. This was more marked among the paediatric population (paediatric vs adult incidence rate ratio in 2019: 3.51 [95% CI 1.12–2.66] vs 1.82 [95% CI 1.05–1.60]). There was an overall increasing rate of AAI prescription for patients admitted for anaphylaxis, but the overall AAI prescription rate was less than 15% and was significantly less likely to be prescribed for the adult compared to paediatric patients (36.5% vs. 89.4%, p < 0.001).

**Conclusions:**

An increasing trend of anaphylaxis incidence rates over the past decade is evident in Asian populations, with a discrepantly low rate of AAI prescription, particularly in the adult patients.

## Background

Anaphylaxis is defined as a potentially fatal, severe and systemic allergic reaction that occurs suddenly after contact with an allergy-causing substance [1]. Although rare, deaths caused by food-induced anaphylaxis are continuing to occur at an estimated rate of 5 to 200 cases per year in the United States [2]. Evidence of changing anaphylaxis incidence has been largely based on the rising trend of hospital anaphylaxis admission rates across time [3]. The burden of anaphylaxis was thought to be lower in Asia and different from the West in terms of varying age distribution, anaphylaxis triggers and low usage of adrenaline auto-injectors (AAI) as first-line treatment [4, 5]. However, a more recent study suggested that children of Asian ethnicity born in Australia may conversely be at higher risk of anaphylaxis compared to other ethnicities [6]. This discrepancy highlights the need for more accurate estimates of the true anaphylaxis burden in Asian countries. Time-trend analyses of anaphylaxis incidence across longer time periods, using a unifying methodology on a territory-wide population, have never previously been reported.

In this study, we took an advantage of a comprehensive electronic records system to determine the incidence rates of anaphylaxis between 2009 and 2019 in Hong Kong and investigated the longitudinal trends of AAI prescriptions.

## Methods

The Hospital Authority (HA) of Hong Kong has established a comprehensive clinical information system with a unified medical record database encompassing more than 7.1 million unique patients across the entire territory. Data were obtained from the Clinical Data Analysis and Reporting System (CDARS)—a centralized electronic database of the HA which captures patients’ data from all public hospitals in the territory. The HA is the sole public-funded healthcare provider, which provides about 90% of in-patient care services across the territory. It serves a population of more than 7 million through 18 Emergency Departments (ED) among the 43 hospitals. These hospitals are organized into 7 clusters based on geographical locations; namely: Hong Kong East, Hong Kong West, Kowloon Central, Kowloon East, Kowloon West, New Territories East (NTEC) and New Territories West Clusters [7, 8]. As around 92% of the population were of Chinese ethnicity, our data likely reflects anaphylaxis in a predominantly Asian population [9].

Data were extracted by a standardized protocol and cross-checked independently by two physicians. All in-patient records between 1st January 2009 and 31st December 2019 with physician-reported diagnosis of anaphylaxis, as classified by the International Classification of Diseases, Ninth Revision (995.0, 995.60–995.69), were extracted from the CDARS, anonymized and analysed. Since CDARS has been built to automatically map the ICD-10 coded diagnoses with that coded by ICD-9, our database search captures all anaphylaxis cases coded by physicians during the study period. Data obtained included patients’ age, gender, admission date, length of stay, and list of prescription medications at time of discharge. Paediatric patients are defined as those less than 18 years of age.

Further subgroup analysis was performed to understand the trends of food allergy diagnoses, co-morbid allergic conditions, anaphylaxis manifestations and accuracy of coded diagnoses. Approval from the Institutional Review Board (IRB) allowed retrieval of data from all paediatric patients of the NTEC from the same period. All patient medical records were reviewed through the electronic records system, including patients’ demographic data, details of allergic reactions, suspected allergens and diagnostic coding. In addition to extracting cases with anaphylaxis, cases with other allergy-related coding including 995.1 (angioneurotic oedema), 995.2 (drug allergy), 995.3 (allergy, unspecified), 708.0, 708.1, 708.8, 708.9 (urticaria) and 995.2 (unspecified adverse effect of unspecified drug, medicinal and biological substance) were also extracted. Each medical record was individually reviewed to evaluate the diagnosis of anaphylaxis in accordance with the National Institute of Allergy and Infectious Diseases/Food Allergy and Anaphylaxis Network criteria [1].

### Statistical analysis

Categorical variables are expressed as number (percentage), and continuous variables are expressed as either mean (standard deviation) or median (range) when appropriate. Univariate and multivariate analyses were used to identify independent associations between demographics and clinical characteristics with AAI prescription. The Chi squared statistic and independent samples *t* test were used to compare categorical and continuous variables between groups in univariate analysis, respectively. Variables with a *P* value of 0.1 or less from univariate analysis were included in multivariate logistic regression to determine which variables were independently associated. A *P* value of less than 0.05 was considered statistically significant for the multivariate analysis. SPSS Statistics version 20 (IBM, Armonk, NY, USA) was used for all analyses. The incidence rates (IR) were calculated by the number of patients divided by the number of total estimated population of Hong Kong between 2009 and 2019. Using the IR of year 2009 as reference, the incidence rate ratios (IRR) were calculated as IR = IR_yi_/IR_y0_, where IR_yi_ refers to the incidence rate of year _i_; IR_y0_ refers to the incidence rate of year 2009. Joinpoint regression using the software provided by Surveillance Research Program of the US National Cancer Institute. The AAI prescription rates were calculated by dividing the number of AAI prescriptions by the number of patients with anaphylaxis. Population statistics from the Census and Statistics Department (Hong Kong Government) were extracted for calculations [10]. Our Census used 19 years old as the cut-off age for paediatric population, thus estimates for the breakdown of paediatric and adult anaphylaxis incidence rates were calculated using population data for < 20 and ≥ 20 years, respectively. This study was reviewed and approved by the IRB of the Joint Chinese University of Hong Kong—NTEC Clinical Research Ethics Committee.

## Results

### More than twofold increase in anaphylaxis incidence between 2009 and 2019

**Fig. 1:**
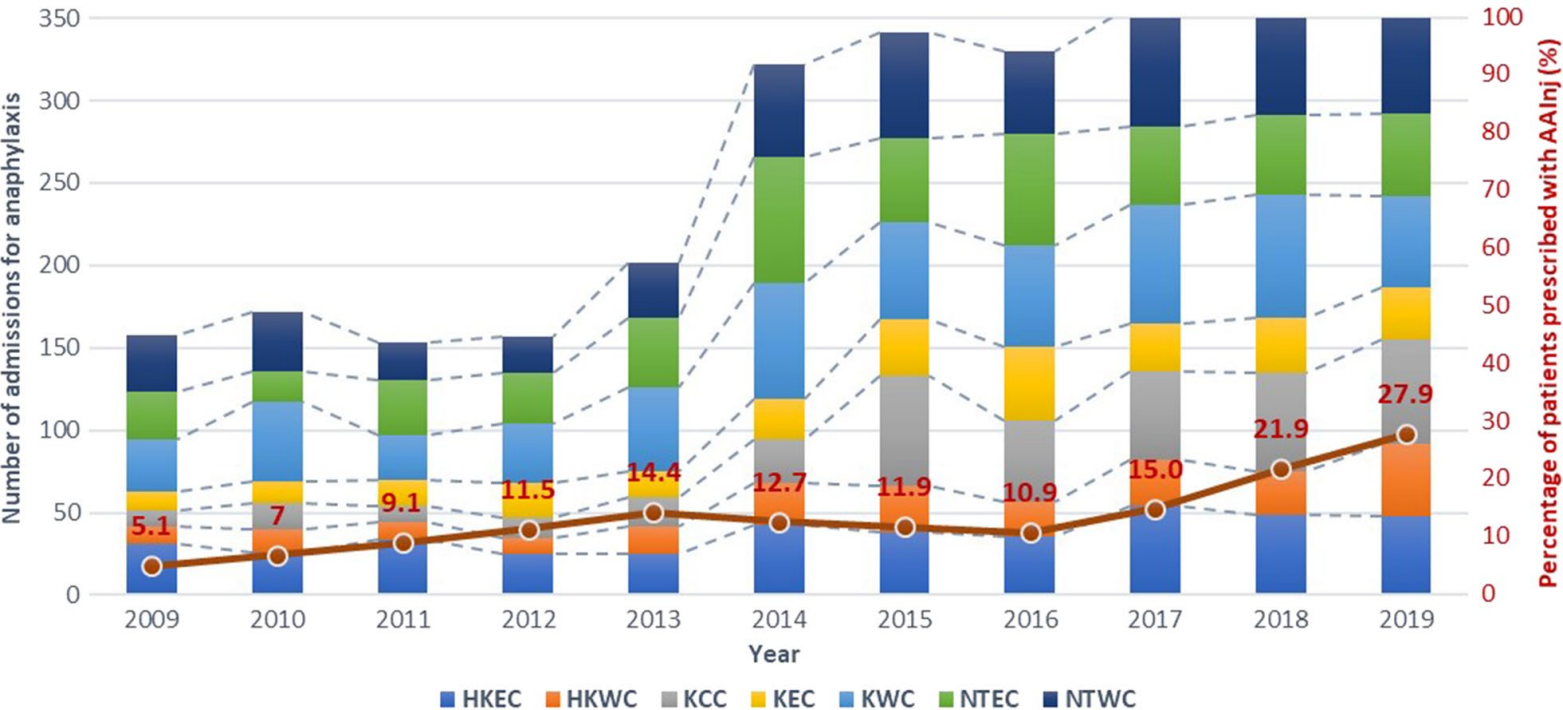
Number of admissions for anaphylaxis (by cluster) and rate of AAI prescription from 2009–2019. *HKE  *Hong Kong East Cluster*, HKWC* Hong Kong West Cluster*, KEC* Kowloon Central Cluster*, KEC* Kowloon Easter Cluster*, KWC* Kowloon West Cluster*, NTEC* New Territories Easter Cluster*, NTWC* New Territories Easter Cluster

Between 2009 and 2019, there were a total of 2,854 patients admitted 2,961 times with a physician-reported diagnosis of anaphylaxis over the span of 11 years. Detailed breakdown of the demographics, admissions and rates of AAI prescriptions of patients per year is shown in Table 1. The number of admissions (per geographical locations) and proportion of patients discharged with AAI per year are displayed in Fig. 1. The overall 10-year estimated incidence rate was 3.57 per 100,000 person-years; the male to female ratio was 0.52 and the median age was 46 years (range 0–98 years). The estimated incidence rates and incidence rate ratios (using year 2009 as reference) per year are shown in Table 2. The increase in the anaphylaxis incidence rates was more than twofold from 2009 to 2019, with a particularly marked increase between 2013 and 2014 (2.80 to 4.44 per 100,000 population, respectively). This significant increase corresponded with an increasing incidence rate ratio of 1.96 (95% CI 1.11–1.62) in 2014 to 2.06 (95% CI 1.13–1.65) in 2019. The increased incidence was also much greater among the paediatric population (paediatric vs adult incidence rate ratio: 2.11 vs 2.30 per 100,000 population in 2009 to 7.40 vs 4.18 per 100,000 population in 2019; paediatric vs adult incidence rate ratio: 3.51 (95% CI 1.12–2.66) vs 1.82 (95% CI 1.05–1.60) in 2019). The increase in anaphylaxis incidence was most marked in the first half of the decade (i.e. from 2009 to 2014), as the incidence risk ratio from 2015–2019 (using year 2014 as reference) did not reach statistical significance (Additional file 1: Table S2).

**Table 1:** Demographics and rates of AAI prescriptions of patients admitted for anaphylaxis from 2009–2019

	2009	2010	2011	2012	2013	2014	2015	2016	2017	2018	2019
Total patients	158	172	153	157	202	322	327	321	340	351	351
Total admissions	164	180	162	161	209	329	341	330	357	362	366
Male gender	92 (58.2%)	106 (61.6%)	91 (59.5%)	78 (49.7%)	108 (53.5%)	159 (49.4%)	157 (48.0%)	149 (46.5%)	171 (50.3%)	178 (50.7%)	184 (52.4%)
Median age (IQR)	47 (38)	45 (32)	45 (31)	46 (32)	49 (35)	45 (32)	47 (1–95)	51 (1-90)	44 (1–98)	45 (1–98)	39 (1–97)
Paediatric (< 18 years)	27 (17.1%)	32 (18.6%)	21 (13.7%)	20 (12.7%)	32 (15.8%)	60 (18.6%)	58 (17.7%)	49 (15.3%)	69 (20.3%)	73 (20.8%)	86 (24.5%)
Adult (≥ 18 years)	131 (82.9%)	140 (81.4%)	132 (86.3%)	137 (87.3%)	170 (84.2%)	262 (81.4%)	269 (82.3%)	272 (84.7%)	271 (79.7%)	278 (79.2%)	265 (75.5%)
AAI prescriptions											
Total patients prescribed AAI	8 (5.1%)	12 (7.0%)	14 (9.2%)	18 (11.5%)	29 (14.4%)	41 (12.7%)	39 (11.9%)	35 (10.9%)	51 (15.0%)	77 (21.9%)	98 (27.9%)
*Paediatric*	7 (25.9%)	8 (25.0%)	10 (47.6%)	9 (45.0%)	15 (46.9%)	32 (53.3%)	30 (51.7%)	21 (42.8%)	35 (50.7%)	46 (63.0%)	55 (64.0%)
*Adult*	1 (0.8%)	4 (2.9%)	4 (3.0%)	9 (6.6%)	14 (8.2%)	9 (3.4%)	9 (3.3%)	14 (5.1%)	16 (5.9%)	31 (11.2%)	43 (16.2%)

**Table 2:** Estimated incidence rate and incidence rate ratios of anaphylaxis from 2009–2019

	2009	2010	2011	2012	2013	2014	2015	2016	2017	2018	2019
Population of Hong Kong [10]	6,966,400	7,052,100	7,109,500	7,171,000	7,210,900	7,252,900	7,309,700	7,377,100	7,413,100	7,486,400	7,500,700
Aged 0–19	1,281,000 (18.4%)	1,257,600 (17.8%)	1,236,900 (17.4%)	1,220,100 (17.0%)	1,194,600 (16.6%)	1,183,300 (16.3%)	1,174,500 (16.1%)	1,167,600 (15.8%)	1,152,800 (15.5%)	1,155,900 (15.4%)	1,161,800 (15.4%)
Estimated incidence rates (per 100,000 population)											
Total incidence rate	2.27	2.44	2.15	2.19	2.80	4.44	4.47	4.35	4.59	4.69	4.68
*Paediatric*	2.11	2.54	1.70	1.64	2.68	5.07	4.94	4.20	5.99	6.32	7.40
*Adult*	2.30	2.42	2.25	2.30	2.83	4.32	4.38	4.38	4.33	4.39	4.18
Incidence rate ratios^a^											
Total incidence rate ratio (95% confidence interval)	*Ref*	1.07 (0.83, 1.28)	0.95 (0.78, 1.22)	0.96 (0.79, 1.23)	1.23 (0.89, 1.35)	1.96 (1.11, 1.62)	1.97 (1.11, 1.62)	1.92 (1.10, 1.60)	2.02 (1.12, 1.64)	2.07 (1.14, 1.65)	2.06 (1.13, 1.65)
*Paediatric (95% confidence interval)*	*Ref*	1.20 (0.65, 1.81)	0.81 (0.51, 1.61)	0.78 (0.50, 1.60)	1.27 (0.66, 1.85)	2.40 (0.93, 2.30)	2.34 (0.92, 2.28)	1.99 (0.84, 2.16)	2.84 (1.01, 2.45)	3.00 (1.04, 2.50)	3.51 (1.12, 2.66)
*Adult (95% confidence interval)*	*Ref*	1.05 (0.81, 1.30)	0.98 (0.78, 1.26)	1.00 (0.79, 1.27)	1.23 (0.87, 1.37)	1.88 (1.07, 1.62)	1.90 (1.07, 1.63)	1.90 (1.07, 1.63)	1.88 (1.07, 1.62)	1.91 (1.08, 1.63)	1.82 (1.05, 1.60)

^a^Incidence rate ratios calculated using year 2009 as reference

### Fewer than 15% of anaphylaxis survivors prescribed AAI and adult patients significantly less likely to have prescriptions

Overall, 14.8% (422/2854) patients admitted for anaphylaxis had prescribed AAI on their medication record prior to discharge. To identify factors associated with AAI prescription, the demographics and clinical demographics (including age, gender, number of admissions, length of stay) were included in univariate analysis. Male gender (59.0% vs 50.3%, *P *= 0.001) and adult age group (36.5% vs. 89.4%, *P *< 0.001) were found to be significant factors in univariate analysis (*P *< 0.10), but other variables including number of admissions and length of stay did not reach statistical significance (data not shown). Further multivariate analysis confirmed that only age group was independently associated with AAI prescription while gender was not. Paediatric patients admitted for anaphylaxis were significantly more likely to be prescribed AAI compared to adult patients (OR = 14.434, 95% CI 11.378–183.310, *P *< 0.001).

### Increasing trend of AAI prescription, especially among adult patients admitted for anaphylaxis

**Fig. 2:**
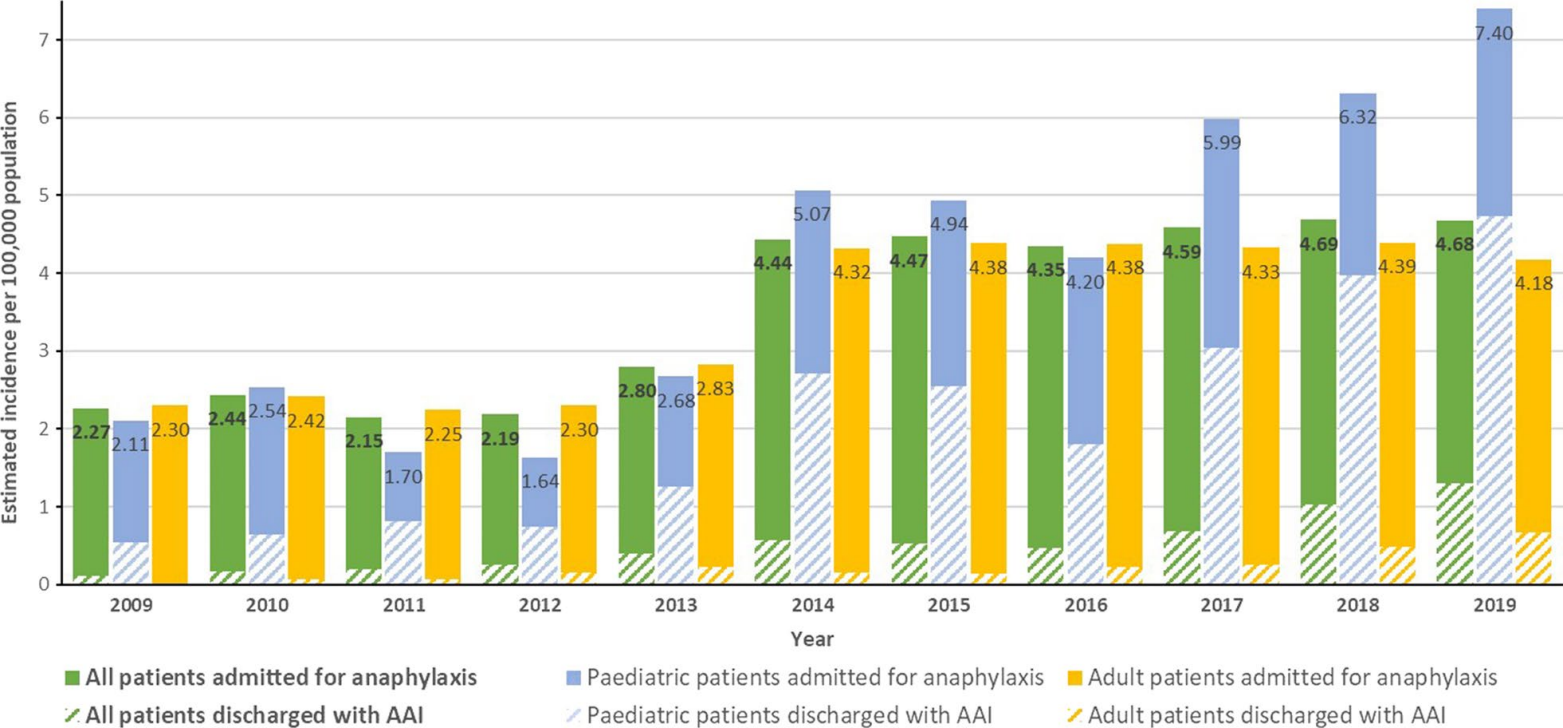
Incidence rates of anaphylaxis (by age group) and rates of AAI prescription from 2009–2019

There was an overall increasing rate of AAI prescription for patients admitted for anaphylaxis during the study period. The AAI prescription rates among adult and paediatric patients with anaphylaxis are displayed longitudinally in Fig. 2. Patients admitted for anaphylaxis in the year 2019 were significantly more likely to be prescribed an AAI compared to those admitted in 2009 (27.9% vs. 5.1%; OR = 7.263, 95% CI 3.436–15.352; *P *< 0.001). This difference was more marked in subgroup analysis of adult patients (16.2% vs. 0.8%; OR = 25.180, 95% CI 3.427–185.020; *P *< 0.001), but also held true for paediatric patients (64.0% vs. 25.9%; OR = 5.069, 95% CI 1.928–13.329; *P *= 0.001) between 2009 and 2019.

### Increased anaphylaxis incidence correlated with the rise in food allergy incidence among the paediatric subgroup

Analysis of the paediatric subgroup from NTEC identified 133 anaphylaxis patients from 2009 to 2019. Review of the diagnostic codes identified 8.1% of misdiagnosed anaphylaxis cases (Additional file 1: Table S1). The median age was 8.3 years and the male:female ratio was 1.2: 1. The majority (58.6%) of patients had other allergic co-morbidities: 16.1%, 27.6%, and 48.3% had concomitant asthma, allergic rhinitis and atopic dermatitis respectively. There was a progressive rise in the incidence of newly diagnosed food allergy from 2009 (12.4 per 100,000 population) to 2019 (38.1 per 100,000 population), which was in line with the increase in anaphylaxis incidence (Additional file 1: Fig. S1). The breakdown of food allergy triggers from 2009 to 2019 is shown in Additional file 1: Fig. S2.

## Discussion

We present a comprehensive longitudinal study of anaphylaxis in Hong Kong over a span of 11 years. With the availability of our territory-wide electronic clinical information system, we were able to calculate the near-absolute anaphylaxis incidence of 3.57 per 100,000 person-years, with an apparent rise in anaphylaxis incidence from 2009 to 2019. In contrast to previous reports, this incidence is comparable to Western populations and we identified a discrepancy of AAI prescription rates between adult and paediatric anaphylaxis survivors.

Although it is difficult to directly compare between studies due to differences in study design and anaphylaxis definitions, our findings are consistent with reports from Western cohorts. For example, the national anaphylaxis data from the UK between 1992 and 2012 found an increase in anaphylaxis admissions from 1 to 7 cases per 100,000 population per annum [11]. The estimated anaphylaxis incidence rates were 1.75 per 100,000 person‐years from the Spanish hospital system during the period 1998–2011 and 1.41 per 100,000 person-years from the Chile’s hospital discharge database between 2001 and 2010 [12, 13]. The incidence rate of anaphylaxis in Olmsted County, Minnesota of the United States was, however, much higher at 42 per 100,000 person-years from 2001 to 2010 [14]. Our novel findings show that Asian populations have also seen a parallel and comparable rise in anaphylaxis incidence to Western cohorts over the past decade. Well‐designed prospective studies using a standardized working definition as well as a unified reporting and collection method of anaphylaxis data are much needed in Asia to better understand how genetic and environmental factors modulate anaphylaxis susceptibility. Identification of potential ethnic- or population-specific modulators may elucidate novel protective or pathomechanisms of anaphylaxis. For example, differences in susceptibility to specific co-factors or adherence to allergen avoidance among different ethnicities have been implicated [15]. Such findings would be invaluable to inform future allergy prevention or treatment strategies both locally and internationally.

Reports on the adherence of AAI prescriptions across different centres and countries. For example, the rates of AAI prescription or retrieval were 54–68% in Olmsted County of the United States; 69.9% in Manitoba, Canada; and 76% in a report from Denmark [16, 17]. In contrast, we identified that fewer than 15% of our anaphylaxis patients were prescribed with AAI. We were also able to confirm that all AAI prescriptions were dispensed and retrieved by patients due to the integration of pharmacies into our public healthcare system. Although there was a gradual improvement in AAI prescription rates (especially in adults) over the past decade, over 70% of patients surviving anaphylaxis in 2019 were still not prescribed with AAI. Since our study only reviewed patients’ discharge medications, the true rate of AAI possession by anaphylaxis patients may be under-estimated as AAI may be prescribed upon subsequent review by allergists. However, as per most international recommendations, AAI should be prescribed for at-risk patients upon discharge from the ED or hospital [18–20]. This is particularly important when there is a time lag between the allergic or anaphylaxis episode and subsequent allergy consultation. The alarmingly low rate of AAI prescription in Hong Kong was, however, worrisome as more than 10% of adult patients with anaphylaxis did not have an identifiable cause and were reported to have lower adherence to dietary avoidance compared to Western cohorts [15]. Our findings therefore heed for an urgent call to improve allergy resources and physician education for anaphylaxis. For example, local or institutional recommendations need to be available and reinforced to optimize the rate of AAI prescription and training among anaphylaxis survivors before discharge. At time of writing, there are still no local guidelines or consensus regarding the prescription of AAI in Hong Kong. It is hoped that findings from this present study will accelerate the dire need to generate local recommendations so that all at-risk patients should also be referred (and timely reviewed) by allergists for accurate diagnosis, evaluation for need of AAI and counselling to prevent recurrent life-threatening episodes in the future.

Our study identified a discrepancy of anaphylaxis care between adult and paediatric patients. During the past decade, paediatric patients were significantly more likely to be prescribed AAI compared to adult patients as shown in our multivariate analysis. In 2009, less than 1% of adult anaphylaxis patients was prescribed an AAI, compared to more than 25% of paediatric patients. Although the rate of AAI prescription subsequently improved for both adult and paediatric patients, only 16% of adult anaphylaxis patients in 2019 had AAI compared to 64% of paediatric patients. We postulate that this may be due to perception of hospital-based physicians that adult patients may be at lower risk of anaphylaxis recurrence due to better allergen avoidance, or lack of local adult allergists [21]. It may also be attributed by the heightened awareness of anaphylaxis in paediatric physicians as allergic diseases, particularly food allergy and eczema, usually occur in the first few years of life [22]. Survivors of anaphylaxis are at continuous risk of repeated life-threatening episodes, with previous studies reporting one in twelve patients experiencing recurrence and one in fifty requiring adrenaline or hospital attention [23]. Food-induced, exercise, and “idiopathic” anaphylaxis have been reported to have even higher recurrence rates [23–25]. Our study highlights the dire demand of allergy services, especially for adult patients presented to ED and hospitals for anaphylaxis.

Our study also noted a sharp increase in anaphylaxis incidence from 2013 to 2014. This coincides with the year with the most marked anaphylaxis fatalities in the United States, and the year when the updated practice parameter for food allergy was issued [2, 26]. Altogether this might have led to the heightened awareness of anaphylaxis in the community and related professions, as well as a shifting behaviour and practice in our patients and health care providers. This demonstrates the importance of continued physician education and promoting anaphylaxis awareness in the community.

The strength of this study is that we used a population-based data set with detailed time-trend, age and sex distribution analyses. The HA’s comprehensive electronic records system also allowed review of all previously prescribed and dispensed medications. Therefore, we were able to ascertain if patients had access to AAI upon discharge, including those who had AAI dispensed prior to index episode of anaphylaxis. However, one of the limitations of this study was the inability to capture information about the anaphylaxis triggers (other than the NTEC subgroup), specific allergy details or calculate symptom severity scores due to the privacy regulations in a deidentified study. Also, data may be incomplete if we identify anaphylaxis triggers based on ICD-9 coding, since causes of anaphylaxis may not be apparent upon initial presentation, but only confirmed after detailed allergy assessment. Our study could not capture patients who do not present to emergency services, but would only be a small proportion and is a limitation common in other studies [27]. Another limitation of this study is that anaphylaxis-related fatalities were not identified/reported, again highlighting the under-recognition of anaphylaxis in our community.

## Conclusion

In conclusion, we report an increase in anaphylaxis incidence between 2009 and 2019 in Asian populations, comparable to the Western world. The increase in anaphylaxis incidence was most marked from 2009 to 2014 and remained stable thereafter. Fewer than 15% of anaphylaxis patients were prescribed with AAI, which was low compared to countries with similar disease burden. AAI was less likely to be prescribed to the adult patients, highlighting the discrepancy in anaphylaxis care between adult and paediatric patients. These findings highlight the urgent need for enhanced allergy education for both hospital-based physicians and family physicians in the community in order to optimize management of anaphylaxis and timely prescription of AAI. Local or regional anaphylaxis registries using standardized anaphylaxis definition, methodology and data collection are in dire need.

## Supplementary information

**Additional file 1: Table S1.** Coding accuracy from 2009-2019 in the subgroup analysis of NTEC paediatric anaphylaxis cases. **Figure S1.** Incidence rates of anaphylaxis and incidence rates of newly diagnosed food allergy from 2009-2019 in the NTEC paediatric subgroup analysis. **Table S2.** Estimated incidence rate and incidence rate ratios of anaphylaxis from 2015-2019, using year 2014 as reference. **Figure S2.** Breakdown of food allergy triggers from 2009-2019 in the NTEC paediatric subgroup analysis.

## Data Availability

The datasets used and/or analysed during the current study are available from the corresponding author on reasonable request.

## Abbreviations

AAI: Adrenaline Autoinjector
PPV: Positive predictive value
HA: Hospital Authority
CDARS: Clinical Data Analysis and Reporting System
ED: Emergency Department

## References

[1] Sampson HA, Munoz-Furlong A, Campbell RL, Adkinson NF, Bock SA, Branum A (2006). Second symposium on the definition and management of anaphylaxis: summary report–Second National Institute of Allergy and Infectious Disease/Food Allergy and Anaphylaxis Network symposium. J Allergy Clin Immunol.

[2] Sampson HA, Aceves S, Bock SA, James J, Jones S, Lang D (2014). Food allergy: a practice parameter update-2014. J Allergy Clin Immunol.

[3] Turner PJ, Campbell DE, Motosue MS, Campbell RL (2020). Global trends in anaphylaxis epidemiology and clinical implications. J Allergy Clin Immunol Pract.

[4] Li J, Ogorodova LM, Mahesh PA, Wang MH, Fedorova OS, Leung TF (2020). Comparative study of food allergies in children from China, India, and Russia: The EuroPrevall-INCO Surveys. J Allergy Clin Immunol Pract..

[5] Tham EH, Leung ASY, Pacharn P, Lee S, Ebisawa M, Lee BW (2019). Anaphylaxis–Lessons learnt when East meets West. Pediatr Allergy Immunol.

[6] Wang Y, Allen KJ, Suaini NHA, McWilliam V, Peters RL, Koplin JJ (2019). The global incidence and prevalence of anaphylaxis in children in the general population: A systematic review. Allergy.

[7] Hospital Authority. Hospital Authority Annual Report 2017–2018 2018 [22/10/2019]. Available from: http://www.ha.org.hk/ho/corpcomm/AR201718/PDF/HA_Annual_Report_2017-2018.pdf.

[8] Authority H. Clusters, Hospitals & Institutions https://www.ha.org.hk/visitor/ha_visitor_index.asp?Content_ID=10036.

[9] Census and Statistics Department. 2016 Population By-census 2016 https://www.bycensus2016.gov.hk/data/16bc-summary-results.pdf.

[10] Census and Statistics Department. Population Estimates 2019 https://www.censtatd.gov.hk/hkstat/sub/so150.jsp.

[11] Turner PJ, Gowland MH, Sharma V, Ierodiakonou D, Harper N, Garcez T (2015). Increase in anaphylaxis-related hospitalizations but no increase in fatalities: an analysis of United Kingdom national anaphylaxis data, 1992–2012. J Allergy Clin Immunol.

[12] Tejedor-Alonso MA, Moro-Moro M, Mosquera González M, Rodriguez-Alvarez M, Pérez Fernández E, Latasa Zamalloa P (2015). Increased incidence of admissions for anaphylaxis in Spain 1998–2011. Allergy.

[13] Hoyos-Bachiloglu R, Morales PS, Cerda J, Talesnik E, González G, Camargo CA (2014). Higher latitude and lower solar radiation influence on anaphylaxis in Chilean children. Pediatr Allergy Immunol.

[14] Lee S, Hess EP, Lohse C, Gilani W, Chamberlain AM, Campbell RL (2017). Trends, characteristics, and incidence of anaphylaxis in 2001–2010: A population-based study. J Allergy Clin Immunol.

[15] Li PH, Thomas I, Wong JC, Rutkowski K, Lau CS (2020). Differences in omega-5-gliadin allergy: East versus West. Asia Pac Allergy.

[16] Lee S, Hess EP, Lohse C, Souza DL, Campbell RL (2016). Epinephrine autoinjector prescribing trends: an outpatient population-based study in olmsted county Minnesota. J Allergy Clin Immunol Pract.

[17] Parke L, Senders AS, Bindslev-Jensen C, Lassen AT, Oropeza AR, Halken S (2019). Adherence to adrenaline autoinjector prescriptions in patients with anaphylaxis. Clin Transl Allergy.

[18] Simons FE, Ardusso LR, Bilò MB, El-Gamal YM, Ledford DK, Ring J (2011). World allergy organization guidelines for the assessment and management of anaphylaxis. World Allergy Organ J.

[19] Boyce JA, Assa’ad A, Burks AW, Jones SM, Sampson HA, Wood RA (2010). Guidelines for the diagnosis and management of food allergy in the United States: report of the NIAID-sponsored expert panel. J Allergy Clin Immunol.

[20] Ewan P, Brathwaite N, Leech S, Luyt D, Powell R, Till S (2016). BSACI guideline: prescribing an adrenaline auto-injector. Clin Exp Allergy.

[21] Lee TH, Leung TF, Wong G, Ho M, Duque JR, Li PH (2019). The unmet provision of allergy services in Hong Kong impairs capability for allergy prevention-implications for the Asia Pacific region. Asian Pac J Allergy Immunol.

[22] Paller AS, Spergel JM, Mina-Osorio P, Irvine AD (2019). The atopic March and atopic multimorbidity: Many trajectories, many pathways. J Allergy Clin Immunol.

[23] Mullins RJ (2003). Anaphylaxis: risk factors for recurrence. Clin Exp Allergy.

[24] Alonso MA, Garcia MV, Hernandez JE, Moro MM, Ezquerra PE, Ingelmo AR (2013). Recurrence of anaphylaxis in a Spanish series. J Investig Allergol Clin Immunol.

[25] Motosue MS, Bellolio MF, Van Houten HK, Shah ND, Campbell RL. Risk factors for recurrent anaphylaxis-related emergency department visits in the United States. Ann Allergy Asthma Immunol. 2018;121(6):717-21 e1. 10.1016/j.anai.2018.08.021 30189249

[26] Dorris S (2020). Fatal food anaphylaxis: Registering a rare outcome. Ann Allergy Asthma Immunol.

[27] Noimark L, Wales J, Du Toit G, Pastacaldi C, Haddad D, Gardner J (2012). The use of adrenaline autoinjectors by children and teenagers. Clin Exp Allergy.

