# Supplementary material for: Increasing incidence of anaphylaxis in Hong Kong from 2009 to 2019—discrepancies of anaphylaxis care between adult and paediatric patients
Source: Clin Transl Allergy. 2020 Nov 19;10:51. doi: 10.1186/s13601-020-00355-6 (PMC7677822; doi:10.1186/s13601-020-00355-6)
Supplement: Supplementary file 1 — Additional file 1: Table S1. Coding accuracy from 2009-2019 in the subgroup analysis of NTEC paediatric anaphylaxis cases. Figure S1. Incidence rates of anaphylaxis and incidence rates of newly diagnosed food allergy from 2009-2019 in the NTEC paediatric subgroup analysis. Table S2. Estimated incidence rate and incidence rate ratios of anaphylaxis from 2015-2019, using year 2014 as reference. Figure S2. Breakdown of food allergy triggers from 2009-2019 in the NTEC paediatric subgroup analysis. [file 13601_2020_355_MOESM1_ESM.docx]

**Additional file 1: Table S1:** **Coding accuracy from 2009-2019 in the subgroup analysis of NTEC paediatric anaphylaxis cases**

| ICD-9 Code | Number of patients | Anaphylaxis upon review but not coded |
| --- | --- | --- |
| 995.0 | 54 | 0 |
| 995.1 | 151 | 29 (19.2%) |
| 995.2 | 86 | 4 (4.7%) |
| 995.3 | 138 | 13 (9.4%) |
| 995.61 | 1 | 0 |
| 708.0 | 27 | 0 |
| 708.1 | 23 | 0 |
| 708.8 | 3 | 0 |
| 708.9 | 258 | 7 (2.7%) |
| **Total** | **718** | **58 (8.1%)** |

**Additional file 1: Table S2:** **Estimated incidence rate and incidence rate ratios of anaphylaxis from 2015-2019, using year 2014 as reference**

|  | **2014** | **2015** | **2016** | **2017** | **2018** | **2019** |
| --- | --- | --- | --- | --- | --- | --- |
| Population of Hong Kong | 7,252,900 | 7,309,700 | 7,377,100 | 7,413,100 | 7,486,400 | 7,500,700 |
| Estimated incidence rates per 100,000 population | 4.44 | 4.47 | 4.35 | 4.59 | 4.69 | 4.68 |
|  |  |  |  |  |  |  |
| Incidence rate ratios* | *ref* | 1.01 | 0.98 | 1.03 | 1.06 | 1.05 |
| (95% confidence interval) | *ref* | (0.86, 1.17) | (0.86, 1.16) | (0.87, 1.18) | (0.88, 1.19) | (0.88, 1.19) |

* Incidence rate ratios calculated usi ng year 2014 as reference

**Additional file 1: Figure S1. Incidence rates of anaphylaxis and incidence rates of newly diagnosed food allergy from 2009-2019 in the NTEC paediatric subgroup analysis**

**
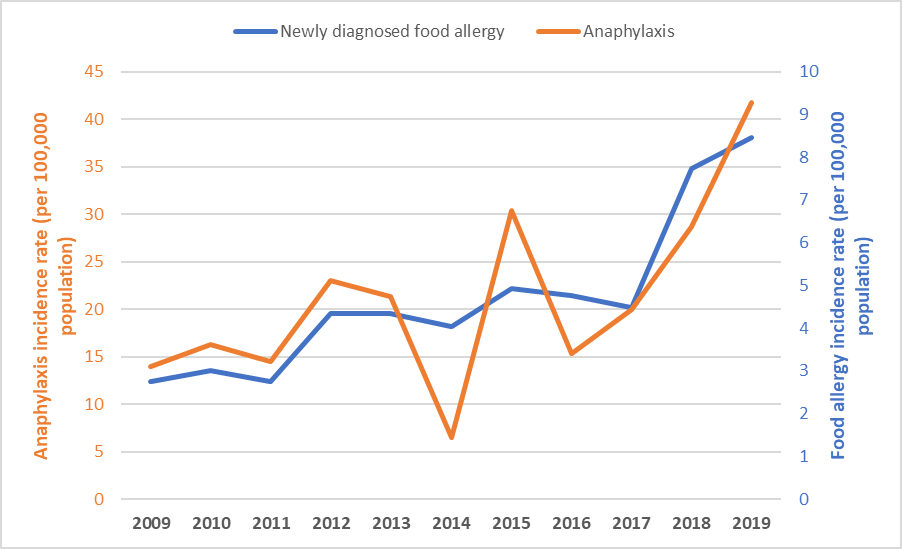
**

**Additional file 1: Figure S2. Breakdown of food allergy triggers from 2009-2019 in the NTEC paediatric subgroup analysis**

**
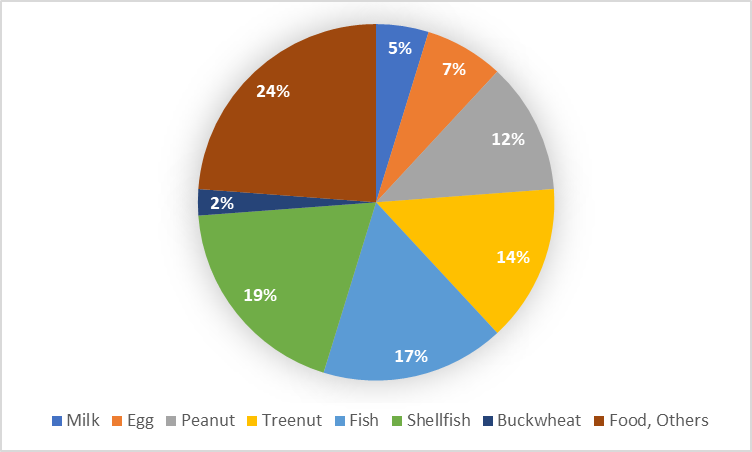
**

Others: bird’s nest, chocolate, grains, Korean jelly, pumpkin seeds, raisins
